# Transthoracic echocardiography performed by intensive care fellows: is minimal focused training enough?

**DOI:** 10.1186/cc9445

**Published:** 2011-03-11

**Authors:** M Almaani, M Alabdrab Alnabi, D Bainbridge, R Taneja

**Affiliations:** 1 University of Western Ontario, London, Canada

## Introduction

Transthoracic echocardiography (TTE) has an important role in the diagnosis of shock in the ICU. There is evidence that noncardiologist residents can address simple clinical questions in the ICU with TTE [1]. We conducted this study to evaluate whether ICU fellows, with minimal focused training in TTE, could reliably acquire good-quality images in critically ill patients.

## Methods

After research ethics board approval, 19 adult patients requiring echocardiography as per the attending physician were enrolled. Patients were enrolled if they were hemodynamically unstable and were adapted on the ventilator. Each patient underwent TTE by one of the certified echocardiographers and then subsequently by a blinded ICU fellow with minimal training in TTE (3-day ultrasound course, 7 hours hands-on training). All images were reviewed offline independently and graded [2] by two blinded reviewers. Interobserver agreement was measured using the intraclass correlation (ICC). Image quality was graded on a scale from 1 (excellent) to 4 (very poor) and the composite image score (total score out of a possible 20 for five views: parasternal short and long axis, apical, subcostal and IVC views) was compared between groups using the Wilcoxin paired test. Each patient's images were further analysed to assess whether the images of LV, RV and IVC had been acquired.

## Results

Nine patients were diagnosed with cardiogenic, eight with distributive and two patients with hypovolemic shock at the time of enrollment in the study. A total of 169 images were analysed. The ICC for interobserver agreement was good (0.8). There was no statistical difference between the composite image scores acquired by ICU fellows (12.3 ± 0.7) (mean ± SE) in comparison with certified echocardiographers (11 ± 0.6, *P *= 0.08). However, the ICU fellows could not acquire images of the RV or LV in five out of 19 patients (26%) in comparison with corresponding images by certified echocardiographers.

## Conclusions

ICU fellows, with minimal focused training in TTE, can acquire images that are comparable in quality with certified echocardiographers in our institution. However, they are not able to acquire images of the LV or RV in over 25% patients as compared with certified echocardiographers. Minimal focused training in TTE may not be enough when managing critically ill patients.
